# Supplementary material for: Comparative Nanopore Sequencing-Based Evaluation of the Midgut Microbiota of the Summer Chafer (Amphimallon solstitiale L.) Associated with Possible Resistance to Entomopathogenic Nematodes
Source: Int J Environ Res Public Health. 2022 Mar 15;19(6):3480. doi: 10.3390/ijerph19063480 (PMC8950650; doi:10.3390/ijerph19063480)
Supplement: Supplementary file 1 [file ijerph-19-03480-s001.zip › ijerph-1602107-supplementary.pdf]

International Journal of *Environmental Research and Public Health*

Supplementary Material

Comparative Nanopore Sequencing-Based Evaluation of the Midgut Microbiota of the Summer Chafer (*Amphimallon solstitiale* L.) Associated with Possible Resistance to Entomopathogenic Nematodes

Ewa Sajnaga [1,*], Marcin Skowronek [1], Agnieszka Kalwasińska [2], Waldemar Kazimierczak [1], Magdalena Lis [1], Monika Elżbieta Jach [3], Adrian Wiater [4]

1 Laboratory of Biocontrol, Production, and Application of EPN, Centre for Interdisciplinary Research, The John Paul II Catholic University of Lublin, Konstantynów 1J, 20-708 Lublin, Poland; marcin.skowronek@kul.pl (M.S.); waldemar.kazimierczak@kul.pl (W.K.); magdalena.lis@kul.pl (M.L.)

2 Department of Environmental Microbiology and Biotechnology, Nicolaus Copernicus University in Torun, Lwowska 1, Torun, 87-100, Poland; kala@umk.pl

3 Department of Molecular Biology, Institute of Biological Sciences, The John Paul II Catholic University of Lublin, Konstantynów1J, 20-708 Lublin, Poland; monika.jach@kul.pl

4 Department of Industrial and Environmental Microbiology, Institute of Biological Sciences, Maria Curie-Skłodowska University, Akademicka 19, 20-033 Lublin, Poland; adrian.wiater@mail.umcs.pl

***Corresponding author**

Ewa Sajnaga e-mail: ewa.sajnaga@kul.pl

Table S1. List of sampling sites

Sample name	Developmental stage of larvae	Geographic origin	EPN treatment (mutualistic bacterial species)
G1, G2, G5, G6, G7	L2	Forest nursery	none
G3, G3R, G4	L3		none
G8, G9, G10, G11, G12	L3	Urban lawn	none
GA1, GA2 GA3	L2	Forest nursery	*Steinernema arenarium* (*Xenorhabdus kozodoii)*
GB1, GB2, GB2R, GB3	L2	Forest nursery	*Steinernema bicornutum* (*Xenorhabdus budapestensis*)
GC1, GC2, GC3	L2	Forest nursery	*Steinernema carpocapse* (*Xenorhabdus nematophila*)
GK1, GK2	L2	Forest nursery	*Steinernema kraussei* (*Xenorhabdus bovienii)*
GM1, GM2, GM3	L2	Forest nursery	*Heterorhabdis megidis* (*Photorhabdus temperata*)

Forest nursery: 51°23'37.0"N 22°29'44.0"E; Urban lawn: 51°23'37.0"N 22°29'44.0"E

Table S2. Proportions of the top twenty most abundant 100% prevailed bacterial genera detected in the midgut of *A. solstitiale* larvae

	Bacillus	*Microbacterium*	*Paenibacillus*	*Turicibacter*	*Bacteroides*	*Anaerotruncus*	*Bradyrhizobium*	*Virgibacillus*	*Rhizobium*	*Cutibacterium*	*Dysgonomonas*	*Ruminococcus*	*Anaerotignum*	*Staphylococcus*	*Acinetobacter*	*Nocardioides*	*Mycolicibacteri*	*Fournierella*	*Corynebacterium*	*Clostridium*
**G10**	2,339	2,051	1,219	0,964	10,272	0,731	5,057	0,030	0,042	3,372	0,013	0,540	0,114	1,039	2,362	1,426	0,534	0,297	2,026	0,224
**G11**	6,058	3,602	0,465	0,029	1,281	0,104	3,068	2,124	5,709	2,943	0,005	0,146	0,007	1,618	16,701	3,373	0,176	0,052	1,937	0,082
**G12**	13,391	0,690	0,301	14,179	1,152	1,359	0,329	7,427	0,306	0,565	0,003	1,132	0,711	0,032	0,002	1,701	1,758	0,811	0,238	2,410
**G1**	0,808	0,051	0,340	0,010	5,552	4,881	0,007	0,236	0,987	0,734	0,002	1,273	1,680	3,481	0,003	0,104	0,003	1,122	0,965	1,062
**G2**	0,082	0,006	0,067	0,081	17,052	14,943	0,003	0,007	0,003	3,611	0,007	3,719	3,411	5,932	2,344	0,137	0,003	3,830	3,965	0,249
**G3R**	3,609	0,008	0,653	2,091	4,069	1,733	0,005	1,542	0,020	0,013	16,204	4,492	3,362	0,013	0,003	0,016	0,014	1,191	0,008	1,585
**G3**	0,484	0,015	1,065	2,174	2,624	3,972	0,010	0,113	0,019	0,009	1,733	4,841	2,101	0,018	0,009	0,004	0,005	2,498	0,008	0,938
**G4**	11,567	0,038	0,359	1,718	0,420	7,497	0,011	5,488	0,027	0,010	3,726	2,037	3,276	0,013	0,051	0,026	0,031	2,071	0,004	0,813
**G5**	1,124	0,009	0,360	0,127	6,532	1,389	6,921	0,020	0,078	6,374	0,005	2,297	3,200	2,096	1,831	1,174	0,499	0,371	3,625	0,653
**G6**	1,900	0,830	4,728	28,383	2,237	0,011	1,763	0,229	0,243	2,856	0,007	0,105	0,225	0,071	0,007	0,894	1,095	0,012	1,995	2,665
**G7**	1,004	0,025	1,265	0,067	4,529	3,161	17,079	0,009	0,635	3,202	0,128	1,347	0,498	0,829	0,006	0,012	3,579	0,593	2,507	0,435
**G8**	0,397	0,363	0,360	0,007	1,130	0,664	5,272	0,005	1,920	1,682	0,002	0,128	0,101	0,892	0,004	1,730	0,437	0,179	0,135	0,137
**G9**	3,053	0,209	0,986	26,691	1,370	0,260	3,753	0,028	0,358	1,573	0,004	0,241	0,063	0,999	0,001	2,343	0,949	0,048	0,103	0,182
**GA1**	0,654	5,026	2,194	0,021	0,323	18,254	1,027	0,009	1,259	0,108	0,001	3,534	0,254	0,172	0,103	0,299	0,346	4,516	0,114	1,492
**GA2**	3,672	1,238	2,541	0,007	2,202	0,256	5,749	0,841	1,055	6,993	0,002	1,328	0,005	5,179	0,002	2,371	1,323	0,048	1,771	0,503
**GA3**	3,703	0,006	1,101	2,998	7,320	1,045	0,002	1,380	0,009	0,984	0,675	0,705	0,497	0,589	0,051	2,512	0,002	0,331	0,361	0,830
**GB1**	2,602	1,587	5,234	0,069	0,497	2,838	7,236	0,203	1,183	0,082	0,001	0,695	0,214	0,005	0,003	0,816	0,744	1,060	0,077	1,375
**GB2**	8,640	0,231	0,421	1,508	7,198	5,118	0,120	4,045	0,106	0,003	12,845	1,418	0,868	0,009	0,002	0,156	0,097	1,362	0,020	1,271
**GB3**	8,564	20,916	26,384	0,462	0,135	0,253	0,548	0,998	1,087	0,006	0,002	0,113	0,314	0,230	0,003	0,319	2,729	0,038	0,012	0,222

**GBR2**	84,227	0,312	1,511	0,006	6,730	0,002	0,119	0,059	0,001	0,003	1,007	0,380	0,002	0,031	0,002	0,001	0,004	0,060	0,433	0,002
**GC1**	0,956	1,984	0,103	0,011	2,746	0,580	2,984	0,123	3,826	1,823	0,003	0,680	0,087	0,402	0,005	2,953	5,510	0,098	0,143	0,133
**GC2**	4,094	0,083	0,405	8,018	0,104	2,292	0,044	3,496	0,044	0,027	0,914	1,897	3,779	0,045	0,676	0,067	0,081	0,407	0,039	1,406
**GC3**	0,200	0,623	0,180	0,010	0,039	2,284	2,061	0,011	0,726	0,007	0,095	0,464	0,507	0,009	0,019	0,516	0,447	0,443	0,010	0,166
**GK1**	40,671	1,058	0,150	0,016	0,725	2,099	0,002	15,925	0,005	0,287	0,004	0,342	0,800	0,181	0,586	0,016	0,137	0,232	0,046	0,058
**GK2**	7,345	30,218	4,651	0,370	0,389	1,742	0,230	0,240	3,891	0,043	0,003	0,395	0,814	0,133	0,033	0,389	0,466	0,260	0,107	0,188
**GM1**	7,948	8,045	19,907	0,038	0,823	0,460	0,840	2,008	5,604	0,769	0,002	0,375	0,525	0,474	0,003	0,430	0,211	0,061	0,265	0,238
**GM2**	10,381	4,310	5,289	0,101	0,241	0,489	2,174	1,232	14,727	0,199	0,005	0,091	0,153	0,113	0,263	0,590	1,292	0,089	0,107	0,088
**GM3**	6,934	20,300	10,119	0,066	0,461	3,416	0,350	1,700	5,558	0,066	0,004	1,026	0,670	0,548	0,002	0,205	0,257	0,506	0,020	0,713

Table S3. Composition of midgut-associated bacteria in the *A. solstitiale* larvae at the genus level

Taxname*	Relative abundance (mean in %) and 95% confidence interval	
	T0 larvae	EPN-tolerant larvae
Turicibacter	5.9 [0.0-12.1]	0.9 [0.0-2.1]
Bacteroides	4.5 [1.6-7.3]	2.0 [0.5-3.5]
Bacillus	3.5 [0.9-6.1]	12.7 [0.5-24.9]
Bradyrhizobium	3.3 [0.4-6.2]	1.6 [0.3-2.8]
Anaerotruncus	3.1 [0.6-5.7]	2.7 [0.2-5.2]
Cutibacterium	2.1 [0.9-3.2]	0.8 [0.0-1.8]
Acinetobacter	1.8 [0.0-4.6]	0.1 [0.0-0.2]
Ruminococcus	1.7 [0.7-2.7]	0.9 [0.4-1.4]
Dysgonomonas	1.7 [0.0-4.4]	1.0 [0.0-2.9]
Anaerotignum	1.4 [0.6-2.3]	0.6 [0.1-1.1]
Virgibacillus	1.3 [0.0-2.8]	2.2 [0.0-4.4]
Staphylococcus	1,3 [0.3-2.4]	0.5 [0.0-1.3]
Nocardioides	1.0 [0.3-1.7]	0.8 [[0.2-1.3]
Paenibacillus	0.9 [0.2-1.7]	5.3 [1.0-9.7]
Rhizobium	0.8 [0.0-1.7]	2.6 [0.4-4.8]
Cloacibacillus	0.6 [0.0-1.3]	1.4 [0.0-3.4]
Microbacterium	0.6 [0.0-1.3]	6.4 [1.1-11.7]
Spiroplasma	0.4 [0.0-0.9]	3.0 [0.0-9.2]
Enterobacter	0.3 [0.0-0.9]	2.9 [0.0-8.9]
Cellulosimicrobium	0.0	2.6 [0.0-5.5]

*Main abundant genera (>1%) are indicated

Table S4. Composition of midgut-associated bacteria in the *A. solstitiale* larvae at the phylum and class level

Taxname*	Relative abundance (mean in %) and 95% confidence interval	
	T0 larvae	EPN-tolerant larvae
Phylum level		
Firmicutes	48.9 [35.6-62.2]	44.0 [30.2-57.8]
Proteobacteria	22.7 [13.8-31.6]	20.0 [9.5-30.6]
Actinobacteria	14.6 [9.1-20.0]	23.5 [14.4-32.7]
Bacteroidetes	7.5 [2.7-12.4]	5.0 [0.0-10.1]
Tenericutes	0.8 [0.2-1.4]	3.4 [0.0-8.1]
Synergistetes	0.7 [0.0-1.5]	1.6 [0.0-3.3]
Class level		
Clostridia	28.8 [17.5-40.1]	18.2 [8.5-28.0]
Bacilli	11.3 [7.5-15.1]	23.4 [10.2-36.7]
α-Proteobacteria	11.2 [5.3-17.1]	13.2 [5.1-21.2]
Actinobacteria	10.8 [6.8-14.9]	22.2 [12.4-32.0]
γ-Proteobacteria	7.8 [3.7-12.0]	5.5 [0.0-14.4]
Bacteroidia	7.3 [2.8-11.9]	4.2 [0.4-7.9]
Erysipelotrichia	7.0 [1.1-12.9]	1.1 [0.0-2.2]
Acidimicrobiia	1.7 [0.2-3.2]	0.5 [0.0-1.1]
Δ-Proteobacteria	1.7 [1.0-2.4]	0.6 [0.3-0.9]
Β-Proteobacteria	1.2 [0.6-1.8]	0.6 [0.1-1.1]
Negativicutes	1.2 [0.2-2.1]	1.0 [0.5-1.5]
Thermoleophilia	1.1 [0.3-1.9]	0.6 [0.1-1.0]
Mollicutes	0.8 [0.3-1.4]	3.4 [0.0-9.7]
Synergistia	0.7 [0.0-1.4]	1.6 [0.0-3.9]

*Main abundant taxa (>1%) are indicated

Table S5. Composition of midgut-associated bacteria in the *A. solstitiale* larvae at the family level

Taxname*	Relative abundance (mean in %) and 95% confidence interval	
	T0 larvae	EPN-tolerant larvae
Ruminococcaceae	12.9 [6.2-19.6]	8.4 [2.2-14.7]
Erysipelotrichaceae	7.0 [0.5-13.5]	1.1 [0.0-2.3]
Lachnospiraceae	6.7 [3.4-10.1]	5.6 [0.5-10.7]
Bacillacea	6.0 [1.4-10.6]	16.2 [2.8-29.5]
Bacteroidacae	4.5 [1.7-7.3]	2.0 [0.5-3.5]
Bradyrhizobiacaeae	4.5 [0.9-8.0]	2.9 [1.0-4.8]
Propionibacteriaceae	2.2 [1.0-3.3]	0.8 [0.0-1.9]
Hungateiclostridiaceae	2.1 [0.6-3.5]	0.8 [0.2-1.4]
Clostridiaceae	1.9 [0.9-2.9]	0.9 [0.4-1.3]
Microbacteriaceae	1.8 [0.5-3.1]	10.0 [3.7-16.3]
Dysgonamonadaceae	1.7 [0.0-4.4]	1.0 [0.0-2.9]
Nocardioidaceae	1.6 [0.6-2.5]	1.1 [0.3-2.0]
Sphingomonadaceae	1.5 [0.7-2.3]	1.0 [0.1-1.9]
Mycobacteriaceae	1.5 [0.0-3.3]	2.0 [0.9-3.1]
Rhizobiaceae	1.1 [0.0-2.3]	5.0 [0.8-9.1]
Paenibacillaceae	1.0 [0.3-1.8]	5.6 [1.1-10.0]
Enterobacteriaceae	1.0 [0.2-1.9]	4.0 [0.0-12.2]
Rikenellaceae	0.9 [0.0-2.5]	1.1 [0.0-2.4]
Synergistaceae	0.7 [0.0-1.5]	1.6 [0.0-3.9]
Spiroplasmataceae	0.4 [0.0-.0.9]	3.0 [0.0-9.2]
Promicromonosporaceae	0.1 [0.0-0.2]	3.5 [0.1-6.9]

*Main abundant families (>1%) are indicated

Table S6. Composition of midgut-associated bacteria in the *A. solstitiale* larvae at the species level

Taxname*	Relative abundance (mean in %) and 95% confidence interval	
	T0 larvae	EPN-tolerant larvae
Turicibacter sanguinis	5.9 [0.0-12.1]	0.9 [0.0-2.1]
Anaerotruncus rubiinfantis	2.4 [0.4-4.1]	2.0 [0.3-3.7]
Cutibacterium acnes	2.0 [0.8-3.1]	0,7 [0.0-1.5]
Bacteroides coprophilus	1.6 [0.0-3.2]	0.6 [0.0-1.2]
Acinetobacter calcoaceticus	1.2 [0.0-3.7]	0.1 [0.0-0.1]
Dysgonomonas gadei	1.2 [0.0-3.0]	0.9 [0.0-2.7]
Fournierella massiliensis	1.0 [0.3-1.7]	0.6 [0.0-1.2]
Bacillus timonensis	0.9 [0.0-1.9]	1.7 [0.0-3.6]
Cloacibacillus porcorum	0.6 [0.0-1.2]	1.3 [0.0-3.2]
Spiroplasma ixodetis	0.3 [0.0-0.8]	2.9 [0.0-9.1]
Enterobacter adburiae	0.0	1.1 [0.0-3.4]
Kaistia defluvii	0.0	1.1 [0.0-2.5]
Microbacterium kyungheense	0.0	2.2]0.0-4.6]
Bacillus pumilus	0.0	3.0 [0.0-9.3]

*Main abundant species (>1%) are indicated

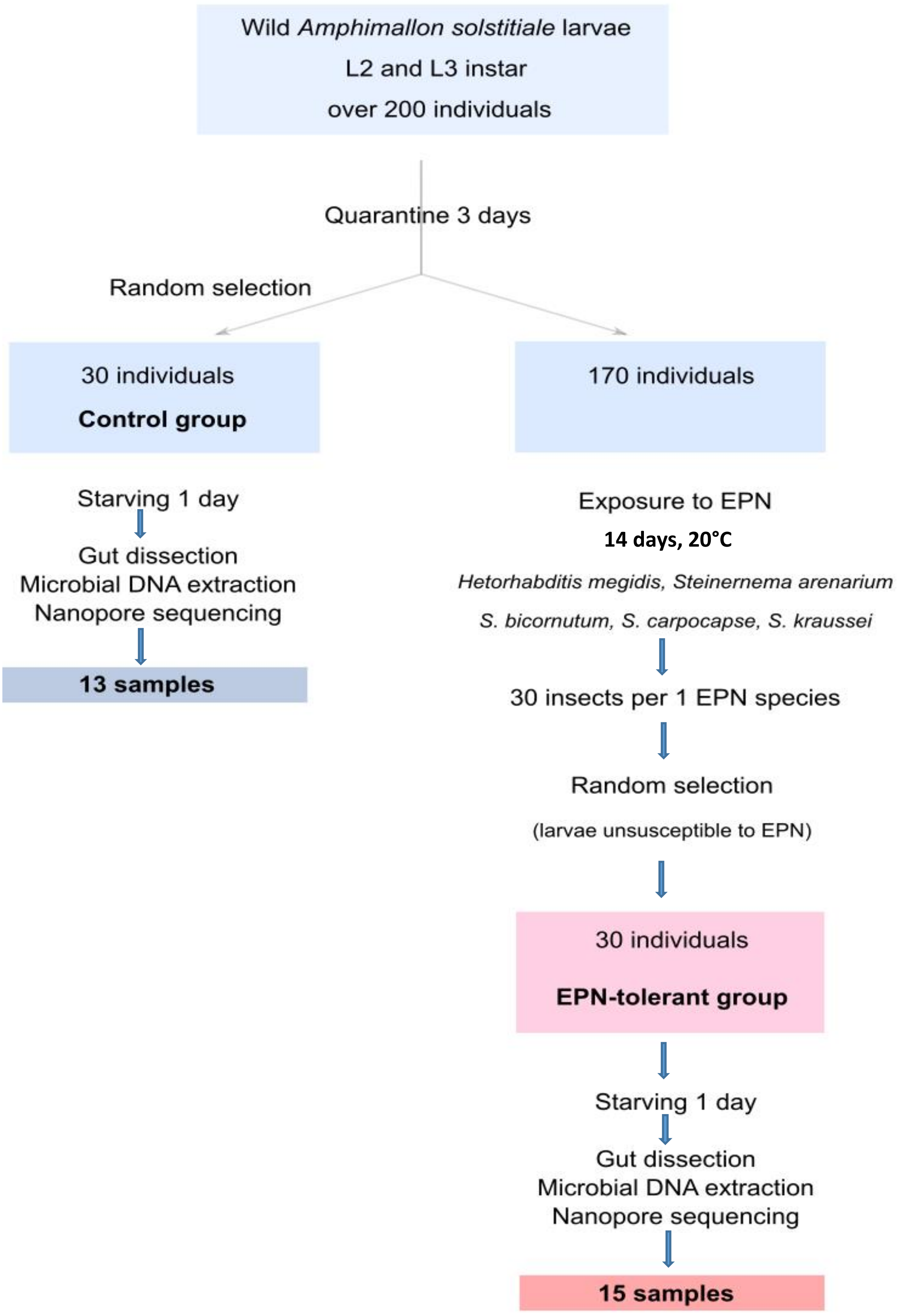

Figure S1. Steps of sample collection and preparation – schematic diagram

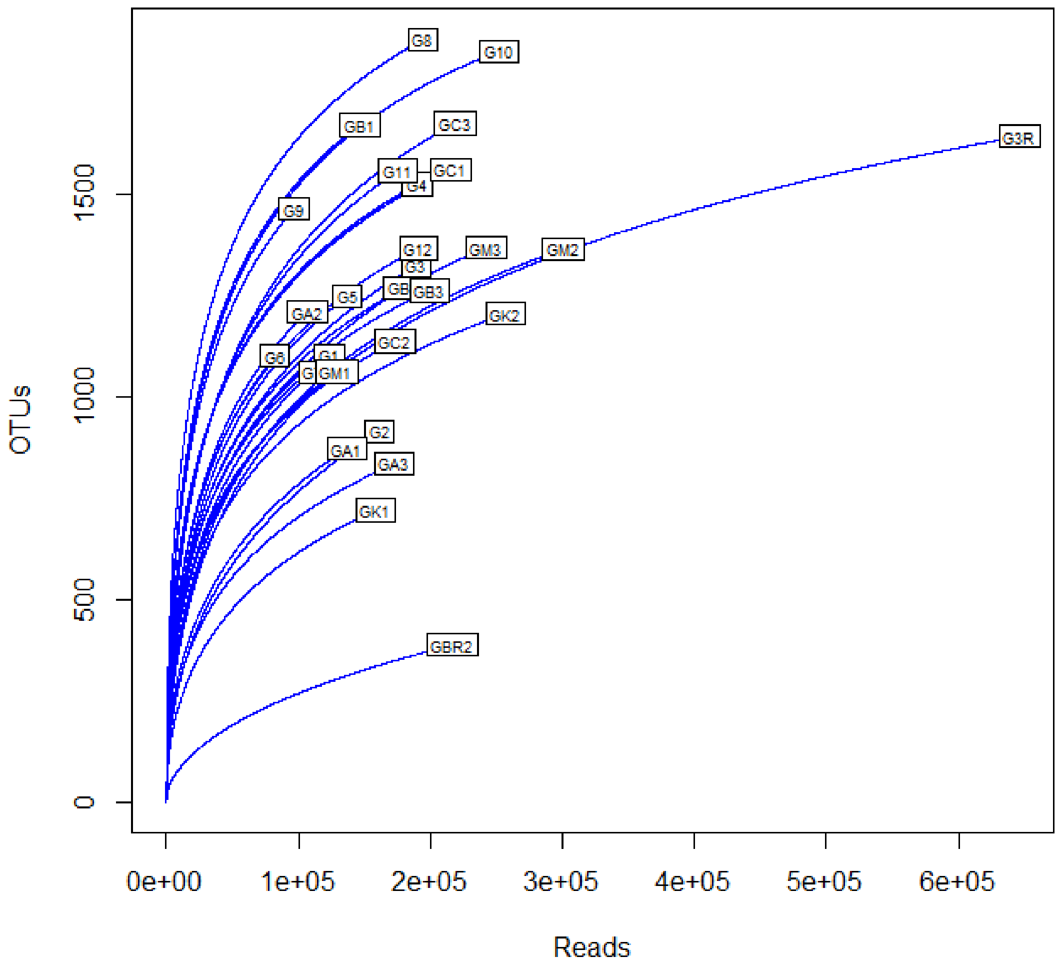

Figure S2. Rarefraction curves from nanopore sequencing of the 16S rRNA gene for all samples.

A.

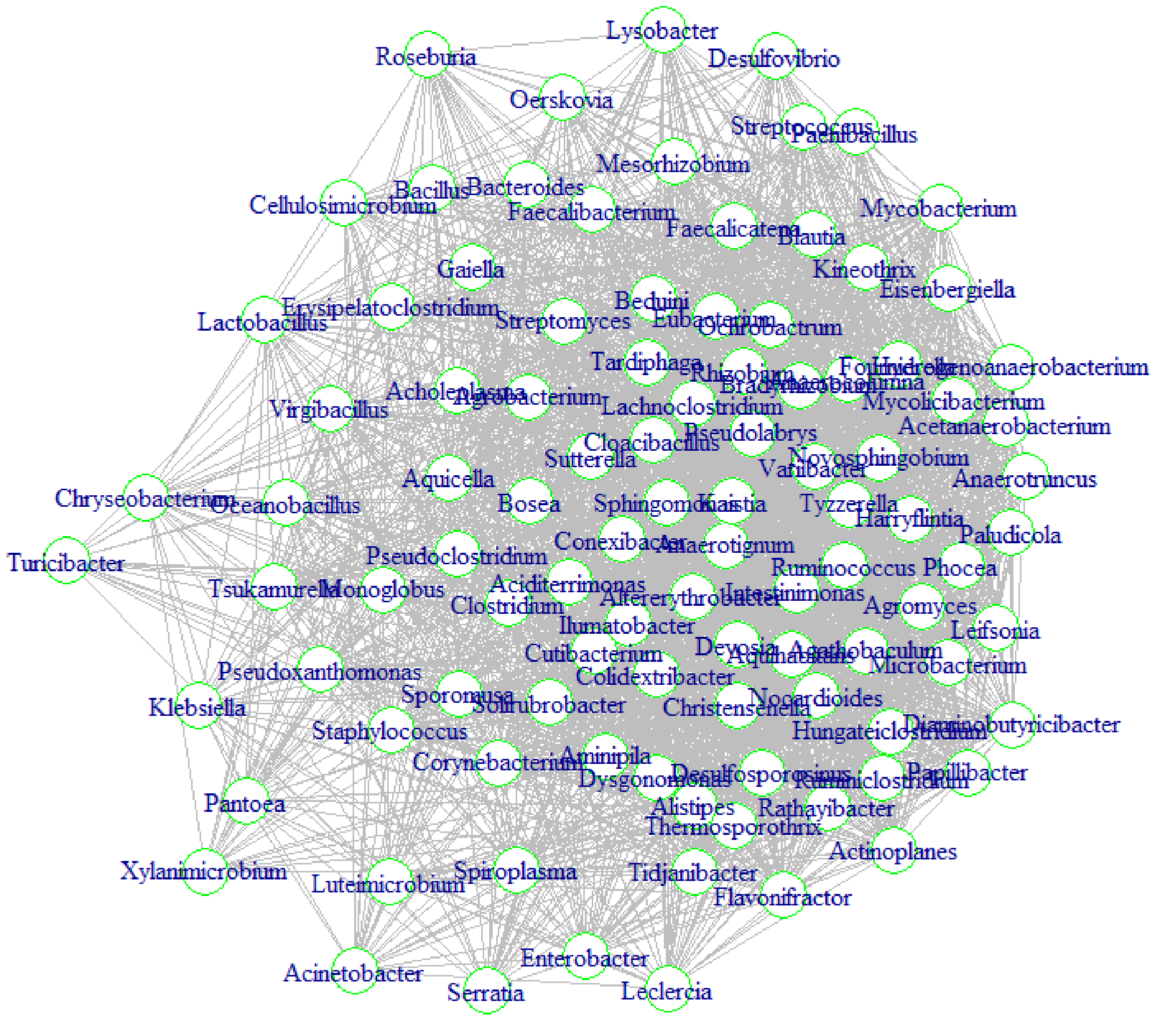

Lysobacter
Roseburia
Desulfovibrio
Oerskovia
Mesorhizobium
Cellulosimicrobium
Bacteroides
Faecalibacterium
Mycobacterium
Faecalicatena
Blautia
Gaiella
Kineothrix
Eisenbergiella
Lactobacillus
Erysipelatoclostridium
Streptomyces
Beduini
Ochrobactrum
Tardiphaga
Virgibacillus
Lachnoclostridium
Mycolicibacterium
Acetanaerobacterium
Pseudolabrys
Cloacibacillus
Sutterella
Aquicella
Novosphingobium
Anaerotruncus
Chryseobacterium
Bosea
Tyzzerella
Harryflintia
Paludicola
Turicibacter
Pseudoclostridium
Anaerotignum
Ruminococcus
Phocea
Clostridium
Intestinimonas
Agromyces
Ilumatobacter
Leifsonia
Cutibacterium
Microbacterium
Pseudoxanthomonas
Colidextribacter
Klebsiella
Solirubrobacter
Christensenella
Nocardioides
Staphylococcus
Corynebacterium
Aminipila
Papillibacter
Pantoea
Alistipes
Rathayibacter
Thermosporothrix
Actinoplanes
Xylanimicrobium
Luteimicrobium
Spiroplasma
Tidjanibacter
Flavonifractor
Enterobacter
Acinetobacter
Serratia
Leclercia

B.

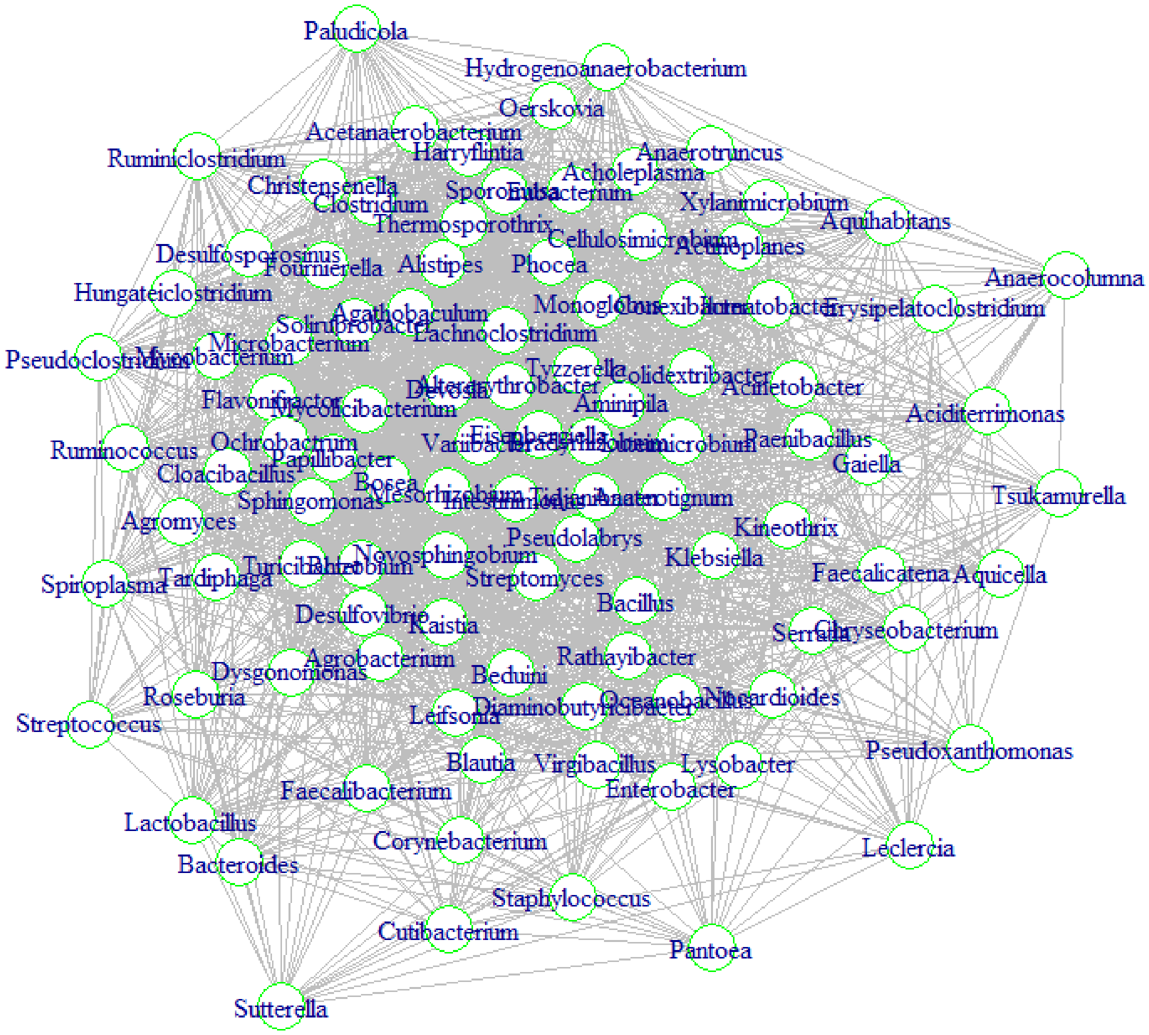

Figure S3. Microbiome networks of *A. solstitiale* midgut bacterial community. A – T0 group; B - EPN-resistant group
